# Can Emergency Medicine Residents Predict Cost of Diagnostic Testing?

**DOI:** 10.5811/westjem.2016.10.31234

**Published:** 2016-11-15

**Authors:** Christopher R. Tainter, Joshua A. Gentges, Stephen H. Thomas, Boyd D. Burns

**Affiliations:** * University of California, San Diego, Department of Emergency Medicine and Department of Anesthesiology, Division of Critical Care, San Diego, California; † The University of Oklahoma, Tulsa, Department of Emergency Medicine, Tulsa, Oklahoma; ‡ Weill Cornell College of Medicine in Qatar and Hamad Medical Corporation, Department of Emergency Medicine, Doha, Qatar

## Abstract

**Introduction:**

Diagnostic testing represents a significant portion of healthcare spending, and cost should be considered when ordering such tests. Needless and excessive spending may occur without an appreciation of the impact on the larger healthcare system. Knowledge regarding the cost of diagnostic testing among emergency medicine (EM) residents has not previously been studied.

**Methods:**

A survey was administered to 20 EM residents from a single ACGME-accredited three-year EM residency program, asking for an estimation of patient charges for 20 commonly ordered laboratory tests and seven radiological exams. We compared responses between residency classes to evaluate whether there was a difference based on level of training.

**Results:**

The survey completion rate was 100% (20/20 residents). We noted significant discrepancies between the median resident estimates and actual charge to patient for both laboratory and radiological exams. Nearly all responses were an underestimate of the actual cost. The group median underestimation for laboratory testing was $114, for radiographs $57, and for computed tomography exams was $1,058. There was improvement in accuracy with increasing level of training.

**Conclusion:**

This pilot study demonstrates that EM residents have a poor understanding of the charges burdening patients and health insurance providers. In order to make balanced decisions with regard to diagnostic testing, providers must appreciate these factors. Education regarding the cost of providing emergency care is a potential area for improvement of EM residency curricula, and warrants further attention and investigation.

## INTRODUCTION

Healthcare expenditures continue to escalate at a significant rate, now representing 17.5% of the gross domestic product (GDP) in the United States. 1 Diagnostic testing is a large proportion of this increase, perhaps prompted by a desire to avoid malpractice claims. 2 This desire must be balanced with cost avoidance to the patient and the healthcare system. Increased awareness of the cost of diagnostic testing may change practice patterns.^3^,^4^

During post-graduate training, emergency medicine (EM) residents learn how diagnostic testing (e.g., laboratory evaluation and radiologic testing) can influence their clinical decision-making. There is a focus on how these tests are interpreted based on current scientific evidence, knowledge of pathophysiology, and emulation of faculty practice patterns. However, often little attention is paid to the potentially detrimental effects of these strategies. In addition to false-positive results, which may lead to unnecessary procedures or additional testing, these tests represent a significant source of resource utilization for the hospital, increased length of stay, and financial burden to the patient and/or health insurance provider.

Needless and excessive spending may occur without an appreciation of the impact on the larger healthcare system. The Accreditation Council for Graduate Medical Education (ACGME) includes cost awareness as a core competency of EM training, although this is recognized as an area of improvement.^5^,^6^ Knowledge of the actual cost of diagnostic testing among EM residents has not been studied, and the specific educational needs in this area are not known.

## METHODS

An anonymous survey was administered to 20 residents from a single ACGME-accredited three-year EM residency program at a hospital-based emergency department (ED) with approximately 60,000 annual visits. The survey consisted of a fill-in-the-blank questionnaire listing 20 commonly ordered laboratory tests and seven radiological exams. It was administered during a single didactic conference during the 2012–2013 academic year. Residents provided their best estimation of the cost of each to the patient. Cost basis was provided by the laboratory billing coordinator and represents patient charges (not institutional cost). Radiology charges did not include a radiologist interpretation fee. We compared responses between residency classes to evaluate whether there was a difference based on level of training. All protocols were reviewed and approved by the institutional review board.

## RESULTS

The survey completion rate was 100% (20/20 residents). There were six postgraduate year- (PGY) 1 (interns), six PGY-2, and eight PGY-3 residents. Twenty-five percent of the residents were female.

We noted significant discrepancies between the median resident estimates and actual charge to patient for both laboratory testing (Table 1) and radiological exams (Table 2) among every residency class, and as a whole. Nearly all estimates were below the actual cost, with only a few estimates above. The group median underestimation for laboratory testing was $114, for radiographs $57, and for computed tomography (CT) exams was $1,058.

The urine drug screen assay was noted to be a particularly expensive test, as it incorporates individual screening tests for eight different drug types: amphetamine/methamphetamines, barbiturates, benzodiazepines, cannabinoids, cocaine, methadone, opiates, and phencyclidine (PCP). Because the order for the test incorporates all of these screens, the charge is far higher than most other laboratory testing studied, even when excluding (as our lab does) further confirmatory testing.

## DISCUSSION

Previous investigations have described a lack of knowledge regarding the cost of testing among internal medicine residents and faculty,^5^,^6^ as well as pediatric residents and faculty, 7 and awareness may be improved with education. 8 There is almost no data to describe if this knowledge deficit exists among EM trainees. The reliance on diagnostic testing in the ED, as well as the escalating costs of providing care make this a particularly important arena in which to improve this knowledge.

Providers cannot make a balanced decision when ordering diagnostic tests without an understanding of costs. Decisions regarding diagnostic evaluation are particularly salient to the ED, where the focus is often diagnosis of undifferentiated complaints. Pursuit of every possible diagnosis in every patient is cost-prohibitive, but cost concerns must be weighed against the possibility of patient harm from missed diagnoses that could require immediate intervention. Charges do not always reflect hospital cost, but they do represent a cost to the healthcare system as a whole. In addition, they may have a significant impact on individuals who do not have the negotiating power of large insurance providers, and may be burdened by the entirety of these charges. Therefore, it is prudent to involve patients in shared decision-making, and this can only be achieved if that information is known by the treating provider.

## LIMITATIONS

This investigation is limited to a single institution and has a small sample size. It reflects similar findings from previous investigations in other specialties. Knowledge about the cost of diagnostic testing is lacking in many EM training programs. However, the improved accuracy demonstrated with increased level of training is encouraging. It is apparent that some degree of familiarity is attained through clinical experience, even if cost estimation is not included as a part of the didactic curriculum.

It is worth noting that the survey results may not reflect the thought process used for clinical decision-making among these residents. Because the participants realized that the aim of the survey was to elicit their knowledge regarding the cost of diagnostic testing, they may have minimized or exaggerated their estimates relative to what they have in mind when ordering a test in the ED. However, it is also likely that the demonstrated lack of awareness may represent the possibility that this does not play a large role in their decision-making process. Finally, it should be noted that the exact charges studied at this institution may not reflect the charges for testing at other institutions.

## CONCLUSION

This pilot study demonstrates that EM residents have a poor understanding of the charges burdened by patients and health insurance providers. In order to make balanced decisions regarding diagnostic testing, providers must appreciate these factors. Education regarding the cost of providing emergency care is a potential area for improvement of EM residency curricula and warrants further attention and investigation.

## Figures and Tables

**Table 1:** Emergency medicine residents’ estimates of laboratory testing charges (U.S. dollars) compared to the actual cost to patients

Test	EM-1 median	EM-2 median	EM-3 median	Group median	Group range	Actual
UA (dip)	20	40	25	25	10–100	60
UA (micro)	50	65	75	50	20–150	71
Type/Screen	60	250	187.5	162.5	10–500	102
CPK	37.5	70	77.5	50	15–300	119
Lipase	37.5	85	50	50	15–300	125
Amylase	27.5	77	62.5	50	15–175	128
ABO/Rh	62.5	175	175	87.5	25–500	135
Rapid strep	25	35	32.5	30	10–200	140
CKMB	37.5	70	100	50	15–300	164
CBC	55	80	107.5	83.5	20–200	166
Trop-I	37.5	75	137.5	50	15–300	174
BNP	37.5	57.5	147.5	55	15–800	185
ABG	30	200	125	100	20–400	185
Blood Cx	85	225	275	200	50–500	198
q-βhCG	57.5	80	100	75	25–300	201
etOH	57.5	67.5	100	87.5	10–250	208
APAP	50	190	150	125	20–800	245
BMP	40	70	100	77.5	20–200	255
CMP	60	97.5	150	110	30–300	274
UDS	62.5	100	225	150	10–500	1136

*BMP*, basic metablic panel; *CMP*, comprehensive metabolic panel; *CPK*, creatine phosphokinase; *CKMB*, creatine kinase-MB; *Trop-I*, troponin-I; *BNP*, brain natriuretic peptide; *q-βhCG*, quantitative β-human chorionic gonadotropin; *UDS*, urine drug screen (qualitative); *etOH*, serum ethanol level; *APAP,* serum acetaminophen level; *Blood Cx*, blood culture; *UA*, urinalysis; *CBC*, complete blood count; *Type/Screen*, blood type and antibody screen; *ABO/Rh*, blood type and Rh type; *Rapid Strep*, group-A strep lateral flow test; *ABG*, arterial blood gas (iStat G7 cartridge).

**Table 2:** Residents’ estimates of radiology testing charges (U.S. dollars) compared to the actual cost to patients

Test	EM-1 median	EM-2 median	EM-3 median	Group median	Group range	Actual charge
pCXR	150	175	175	175	50 – 500	200
Ankle radiograph	150	400	100	150	50 – 750	200
Humerus radiograph	150	275	112.5	137.5	69 – 500	300
CT c-spine	1500	750	800	800	300 – 3500	1700
CT chest	1750	1000	1750	1050	400 – 5000	1800
CT brain	1125	825	1150	950	80 – 4500	2000
CT abd/pel	2500	1000	1750	1257.5	400 – 10000	3000

*pCXR*, portable chest x-ray; *EM,* emergency medicine; *CT,* computed tomography
